# Peptide candidates for the development of therapeutics and vaccines against β-coronavirus infection

**DOI:** 10.1080/21655979.2022.2060453

**Published:** 2022-04-07

**Authors:** Rounak Chourasia, Srichandan Padhi, Loreni Chiring Phukon, Md Minhajul Abedin, Ranjana Sirohi, Sudhir P Singh, Amit Kumar Rai

**Affiliations:** a Institute of Bioresources and Sustainable Development (DBT-IBSD), Regional Centre, Tadong- 737102, India; b Department of Chemical and Biological Engineering, Korea University, 145, Anam-ro, Seongbuk-gu, 02841, Republic of Korea; c Centre of Innovative and Applied Bioprocessing (DBT-CIAB), Sector-81, S.A.S. Nagar, Mohali- 140306, India; d Institute of Bioresources and Sustainable Development (DBT-IBSD), Mizoram Node, Aizawl, India

**Keywords:** Β-CoV, COVID-19, peptides, vaccines, therapeutics

## Abstract

Betacoronaviruses (β-CoVs) have caused major viral outbreaks in the last two decades in the world. The mutation and recombination abilities in β-CoVs resulted in zoonotic diseases in humans. Proteins responsible for viral attachment and replication are highly conserved in β-CoVs. These conserved proteins have been extensively studied as targets for preventing infection and the spread of β-CoVs. Peptides are among the most promising candidates for developing vaccines and therapeutics against viral pathogens. The immunostimulatory and viral inhibitory potential of natural and synthetic peptides has been extensively studied since the SARS-CoV outbreak. Food-derived peptides demonstrating high antiviral activity can be used to develop effective therapeutics against β-CoVs. Specificity, tolerability, and customizability of peptides can be explored to develop potent drugs against β-CoVs. However, the proteolytic susceptibility and low bioavailability of peptides pose challenges for the development of therapeutics. This review illustrates the potential role of peptides in eliciting an adaptive immune response and inhibiting different stages of the β-CoV life cycle. Further, the challenges and future directions associated with developing peptide-based therapeutics and vaccines against existing and future β-CoV pathogens have been discussed.

## Graphical Abstract

**Figure:**
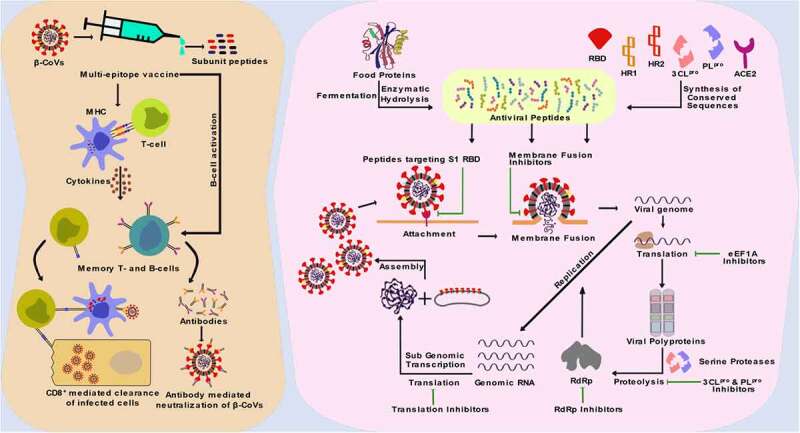

## 1. Introduction

The increase in the emergence and re-emergence of viral respiratory diseases in recent times has gravely threatened public health and the global economy. In the last twenty years, four major viral outbreaks have been recorded, including the highly pathogenic severe acute respiratory syndrome coronavirus (SARS-CoV) [1] and Middle East respiratory syndrome coronavirus (MERS-CoV) [2]. Since December 2019, a highly contagious novel coronavirus, SARS-CoV-2, has been responsible for a respiratory illness called the coronavirus disease of 2019, i.e. COVID-19 [3,4]. A very high basic reproduction number (R0) of 2–2.5 caused an unprecedented spread of the SARS-CoV-2 virus globally [5]. The COVID-19 disease has claimed millions of lives worldwide, becoming the first documented coronavirus pandemic in history [6]. The occurrence of several variants of concern (VOCs), including Alpha (B.1.1.7), Beta (B.1.351), Delta (B.1.617.2), and Omicron (B.1.1.529), has resulted in new waves of SARS-CoV-2 infections, causing considerable loss of lives and economic standstill throughout the world [7].

**Figure 1.**
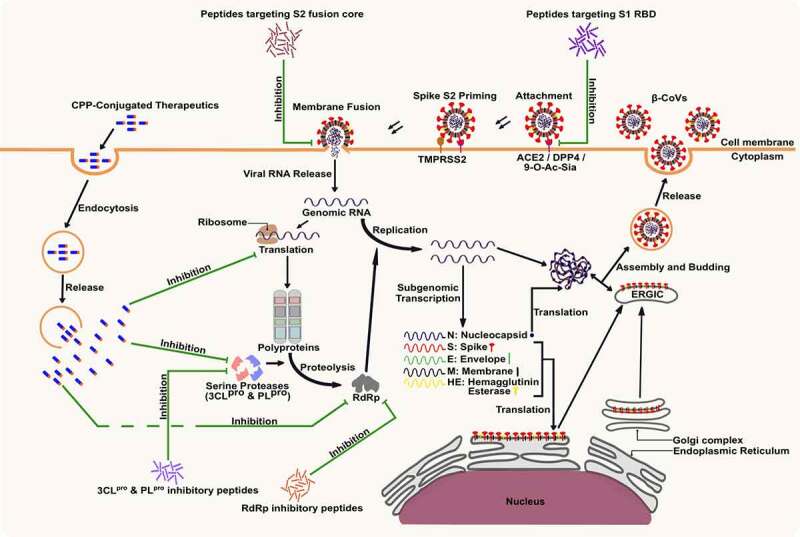
β-CoV lifecycle and potential inhibitory roles of peptides. The targets for the inhibition of β-CoV infection and proliferation include the viral spike protein which binds to the host cell receptors and facilitates entry of the virus into cell cytoplasm, the host translational machinery that is used for the synthesis of viral polyproteins, the viral serine proteases that process the release of viral structural proteins and enzymes, and the viral RNA dependent RNA polymerase (RdRp) enzyme that facilitates the replication of viral genomic RNA.

SARS-CoV and SARS-CoV-2 belong to the B (Sarbecovirus) lineage of the β-CoV genus, while MERS-CoV is the first C (Merbecovirus) lineage β-CoV that can infect humans [8]. Human coronavirus OC43 (HCoV-OC43) and HCoV-HKU1 belong to A (Embecovirus) lineage of β-CoV and cause symptoms of the common cold in humans [9]. SARS-CoV-2 shares 96.2% nucleotide sequence identity with the bat CoV RaTG13, proposing a bat origin of the virus [10,11]. Besides, SARS-CoV-2 shares about 79% and 50% sequence identity with SARS-CoV and MERS-CoV, respectively [11]. Host cell entry and replication mechanisms of all humans infecting β-CoVs are quite similar (Figure 1). The SARS-CoV infection of 2002 resulted in a fatality rate of 11%, whereas the MERS-CoV outbreak in 2012 had a fatality rate of 34% [12]. The fatality rate of SARS-CoV-2 was lower at 1.6%; however, the highly infectious nature of the virus caused it to spread around the world [13] rapidly. The (+) ve sense single-stranded RNA (ssRNA) genome of SARS-CoV-2 enables the virus to be easily detected by the intracellular toll-like receptors (TLRs), which have an affinity towards virus-associated molecular patterns (VAMPS) [14]. The human TLR4 act as the native immune sensor for β-CoV spike proteins and activates several signaling cascades that engender a cytokine storm. This results in uncontrolled inflammation combined with direct virus-induced multi-organ damage, including acute respiratory distress syndrome (ARDS), leading to possible death [12]. New variants of SARS-CoV-2 with increased host cell binding affinity are emerging rapidly, making it difficult to curtail the spread of the virus and end the pandemic [12]. Structural and non-structural proteins of β-CoVs play a crucial role in the virus<apos;>s attachment, replication, and proliferation and thus are promising targets for the inhibition of β-CoV infection [15–18]. Effective treatments are essential to combat β-CoV infections. The development of vaccines and antiviral drugs is critical to alleviating the health and economic burden of diseases caused by β-CoVs [11].

Due to high specificity, efficiency, tolerability, and safety, peptides have increased interest in pharmaceutical research and development (R & R&D) [19]. The use of natural and synthetic peptides to develop novel therapeutics promises the potential to treat various diseases [20]. Diverse sources of therapeutic peptides include microorganisms, plants, food, host defence antimicrobial, and antiviral peptides. Peptides can also be synthesized via recombinant and chemical methods [20–24]. Food-derived antiviral peptides can play an essential role in improving the immune system<apos;>s ability to combat pathogens, thereby enabling individuals to combat pandemic outbreaks without the risk of side effects [25]. Besides, antiviral peptide enriched functional foods can provide nutrition to and ensure the well-being of the people of all ages in a struggling global economy, as required by Goal 3 (Good Health and Well-being) of the United Nations (UN) sustainable development goals (https://www.un.org/sustainabledevelopment/health/) [26,27]. Several studies have reported the ability of natural and synthetic peptides to interact with critical viral proteins, indicating the potential use of these peptides in developing antiviral therapeutics [22,28].

Peptide-based vaccines containing multiple conserved immunodominant epitopes can provide broad immunity against multiple serotypes of a virus [29]. The development of such a vaccine is of high significance as the genetic analysis of SARS-CoV-2 from different countries has revealed the diversification of the virus into several clades and the emergence of VOCs [11]. Additionally, cell-penetrating peptides (CPPs) can cross the cell membrane and carry small therapeutic molecules into cells [30]. In the present review, we discuss the different biotechnological approaches in developing peptide-based antiviral therapeutics that can be explored to combat existing β-CoV infections and similar life-threatening viral diseases in the future.

## 2. Potential therapeutic targets for combating the replication and transmission of β-CoVs

β-CoVs are enveloped, positive-sense, ssRNA viruses with a genome size of approximately 30 kb [11]. The genetic information for NSPs is encoded in two large open reading frames (ORFs), ORF 1a and ORF 1b, which amounts to two-thirds of the β-CoV genome. The remaining one-third of the genome encodes for the structural spike, envelope, membrane, and nucleocapsid proteins [11]. The genome of HCoV-OC43 and HCoV-HKU1 encode for an additional protein, hemagglutinin esterase (HE) [31]. HE functions in viral attachment by specific receptor binding determinant salicylic acids and cleaving off specific O-acetyl groups [32]. β-CoVs are zoonotic pathogens that have crossed the species barrier to infect humans [33]. The origin of SARS-CoV, MERS-CoV, and SARS-CoV-2 can be traced back to bats [31,34–36], while HCoV-OC43 and HCoV-HKU1 have originated from rodents [37–39]. Cross-species transmission of β-CoVs facilitated by direct or indirect zoonotic contacts and sufficient genomic recombination results in the spread of β-CoVs in humans, eventually threatening the emergence of a novel viral disease [31].

The trimeric spike protein is required for the critical function of attachment and fusion of the virus to the host cell-specific receptors. Mutations in β-CoV genomes during the evolution of the virus enable them to infect the human host, importantly by modifying the receptor specificity of the spike protein [33]. The protein receptors in humans for β-CoVs entry are cell surface peptidases, including the angiotensin-converting enzyme 2 (ACE2) for SARS-CoV and SARS-CoV-2 [15,40], dipeptidyl peptidase 4 (DPP4) for MERS-CoV [8], and 9-O-acetylated sialic acid (9-O-Ac-Sia) containing glycan-based receptors for the entry of HCoV-OC43 and HCoV-HKU1 [9]. The binding of the spike protein with host receptors triggers the pathogenesis of β-CoVs (Figure 1). Recent studies have reported that the binding of SARS-CoV-2 spike protein with ACE2 leads to the activation of TLRs, resulting in the release and proliferation of pro-inflammatory cytokines, leading to inflammation [14]. β-CoV spike protein demonstrates a binding affinity with extracellular domains of TLRs (TLR 1, TLR 4, and TLR 6). TLR 4 has the strongest binding affinity to SARS-CoV-2 spike protein, followed by TLR 6 and TLR 1, suggesting the spike protein-TLR 4 interactions to be responsible for the immunological manifestation of SARS-CoV-2 infection [41]. Therapeutics targeting TLRs can be helpful in preventing the spread of β-CoVs and inhibiting the inflammatory response of the host immune system to β-CoV infection. TLR-agonists can be used for pre-stimulation of the host<apos;>s immune system to boost immunity against infection in uninfected individuals. In contrast, TLR-antagonists can be used to prevent the development of cytokine storms and inflammation in individuals infected with β-CoVs by inhibiting the binding of viral spike protein with TLRs [42].

It is reported that the fatality rate of SARS-CoV-2 (1–1.6%) is far lower than that of both SARS-CoV (11%) and MERS-CoV (34%) [43]. However, receptor affinity analysis revealed that SARS-CoV-2 binds to the ACE2 receptor much more efficiently than SARS-CoV, highlighting the highly infectious nature of the novel virus [18,44]. In humans, receptors for β-CoVs are highly expressed in multiple organs, including the kidney, small intestine, liver, and testis, increasing an individual<apos;>s vulnerability to such infections [45]. Interestingly, conserved residues exist in the receptor-binding domain (RBD) of the β-CoV S1 subunit, and inhibitors that competitively bind to these conserved residues of RBD could efficiently block the attachment of the virus to the host receptors (Figure 1) [11]. Following the attachment of S1 protein to the host receptor, a conformational change of the viral S2 protein is triggered, leading to fusion of the viral envelope with the receptor cell membrane [46]. The heptad repeat 1 (HR1) and HR2 domains of the S2 protein play a significant role in β-CoV fusion with the target cell and fusion inhibitory peptides. These conserved domains could be targeted to impede the release of the viral genome inside the host cell cytoplasm (Figure 1) [38]. Due to its critical role in viral attachment, fusion, and entry into host cells, the S protein of β-CoVs has been the primary target for developing therapeutics, including entry inhibitors, antibodies, and vaccines [11].

Replication of the β-CoV RNA is preceded by the translation of the replicase genes from the virion genomic RNA and assembly of the replicase complexes [18]. The viral proteases, e.g. 3CL^pro^ (NSP5), and PL^pro^ (NSP3), are responsible for the processing of viral polyprotein into NSPs, including the RdRp complex (Figure 1) [47–50]. These proteases are excellent targets for inhibiting viral replication as their cleavage specificity is unlike that of any known human proteases [11]. Proteins of the RdRp complex are translated from ORF 1a and ORF 1b into RdRp (NSP12), which in complex with cofactors, NSP7 and NSP8, catalyzes the replication of viral genomic RNA [18]. NSP13, a helicase, is another essential replication enzyme that plays a critical role in the tropism and virulence of β-CoVs. NSP13 can be used as a therapeutic target for inhibiting viral replication [48,51]. Impeding the activity of NSPs could inhibit the replication and proliferation of β-CoV, making these proteins promising targets for the development of inhibitor therapeutics [52,53].

High infection rates lead to genetic mutations in the pathogen resulting in the emergence of variants with increased infectivity and evading immune systems [54]. Since the onset of the COVID-19 pandemic, the emergence of VOCs has been associated with increased transmissibility and enhanced virulence. All the currently reported SARS-CoV-2 VOCs have mutations in the RBD and the N-terminal domain (NTD), increasing the affinity of the viral spike protein to the ACE2 receptor [55]. The Alpha variant includes spike protein changes, including deletion 69–70, P681H, S982A, N501Y, deletion 145, D614G, D1118H, T716I, and A570D [54]. In individuals infected with the B1.1.7 variant, the risk of death was reportedly higher than early SARS-CoV-2 infections [56]. Eight mutations in the S protein, including A701V, D215G, D80A, E484K, K417N, L18F, N501Y, and R246I, led to the emergence of the Beta variant with increased binding affinity for the ACE2 receptors [57]. The Delta variant was initially identified in December 2020 and was responsible for the deadly second wave in India. This variant quickly became the most dominant SARS-CoV-2 VOC globally and is associated with 10 mutations in the spike protein, which caused this variant to have a superior rate of transmission and infections compared to other previously known ones SARS-CoV-2 variants [54]. Due to more than 30 mutations in the S protein, which resulted in a sharp increase in infection cases, the Omicron variant was quickly recognized as a VOC [58]. The *in silico* studies have suggested that the Omicron variant is ten-fold more contagious than the original virus or around twice as infectious as the Delta variant [59]. Three-dimension structure-based analyses of Omicron RBD-antibody interaction have indicated that the B.1.1.529 variant may be twice as likely to escape current vaccines as compared to the Delta variant [59]. A complete experimental analysis of the Omicron variant is necessary and understanding the effects of Omicron infection will take several weeks or even months. The emergence of new SARS-CoV-2 variants challenges the progress made in halting SARS-CoV-2 infections despite the development of vaccines against COVID-19 and mass vaccination efforts. The development of vaccines and therapeutics with potent activity against constantly mutating β-CoVs is necessary to curb the spread of such pathogens.

## 3. Development of peptide-based vaccines and other immunotherapeutics against β-CoV infections

Chemotherapeutic and immunotherapeutic strategies have been proposed for prophylaxis against β-CoV infections and to treat the diseases’ different conditions [60]. Chemotherapy involves the use of different drugs that prevent the spread of infection in the host by inhibiting critical stages such as adhesion, entry, and replication of the virus [60]. Drugs such as Remdesivir, Ivermectin, Heparin, and Camostat Mesylate are some of the chemotherapeutics currently being studied to inhibit SARS-COV-2 infection [60]. However, there is a lack of evidence for curing β-CoV infections by chemotherapy and immunotherapy that helps to control SARS-CoV-2 infection [60]. Immunotherapy involves the use of immunogenic compounds that interact with the host immune system to control the spread of the pathogen and prevent inflammatory responses such as cytokine storms. Immunotherapeutic strategies include vaccination and the use of immunomodulatory agents such as monoclonal antibodies, immunostimulants, and immunosuppressants [60].

**Figure 2.**
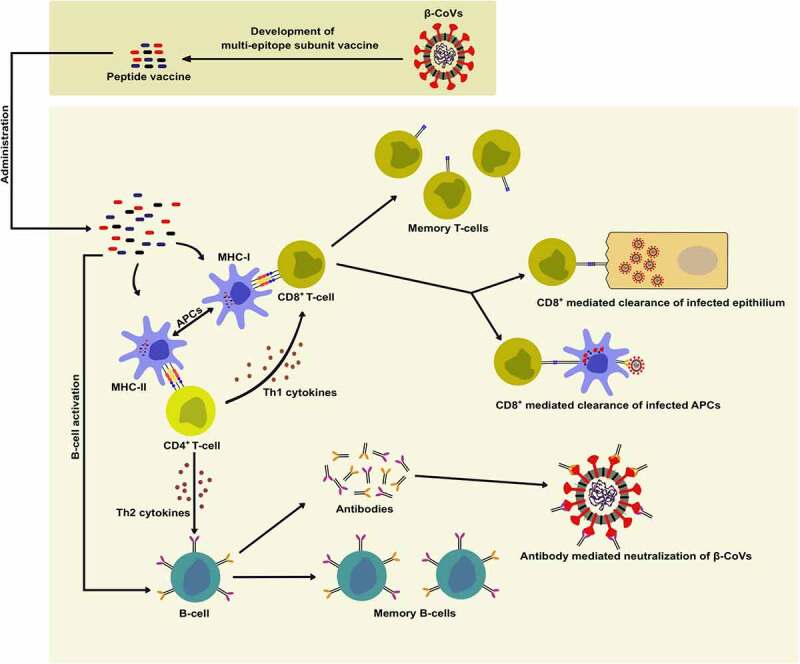
Potential role of peptide-based multi-epitope subunit vaccines in eliciting an adaptive immune response against β-CoV. Peptides can be presented by the major histocompatibility complex (MHC) molecules as antigenic B- and T-cell epitopes which can elicit the clearance of infected epithelium and antigen-presenting cells (APCs), formation of antibodies for the neutralization of viral particles, and result in the generation of memory B- and T-cells.

Vaccines are among the most potent candidates for disease prevention that elicit a memory immune response against the pathogen [24]. Vaccines have successfully been used to prevent several viral pathogens, including pox virus, measles virus, mumps virus, and rubella virus [24]. Among the various types of vaccines, subunit vaccines present several advantages over other vaccines, such as the absence of virulent factors and a relatively safe profile [61]. Additionally, antibodies elicited against inactivated whole-virion or full-length viral structural protein vaccines may lead to antibody-dependent enhancement (ADE), which results in increased viral infection of cells expressing Fc receptors [62]. The development of peptide vaccines can prevent the risk of ADE where synthetic peptides can be used as antigenic B- and T-cell epitopes for the development of subunit vaccines against β-CoVs. Conserved viral peptides can be presented by the major histocompatibility complex (MHC) molecules leading to an adaptive immune response (Figure 2) [63].

The vital function of viral structural proteins to fuse and enter the host cells has attracted several studies on vaccine and antiviral drug development [64]. The host receptor explicitly recognises the S1 RBD subunit of the spike protein, and its sequence is conserved in the downstream C-terminal domain (CTD) of the spike protein of most β-CoVs, including SARS-COV-2, SARS-CoV, HCoV-HKU1, and MERS-CoV [64]. HCoV-OC43 is the only known human infecting β-CoV with the RBD present in the NTD of the spike protein [65]. Similarly, the N protein of β-CoVs is a highly conserved and antigenic structural protein with multiple functions, including nucleocapsid formation, signal transduction, RNA replication, and mRNA transcription [66]. The conserved nature and critical function of β-CoV S and N protein could be a breakthrough in vaccine development.

A recent study has identified a set of highly conserved B- and T-cell epitopes in SARS-CoV S and N proteins that can be used for designing vaccines against SARS-CoV-2 [67]. Administration of SARS-CoV S1 and N protein fragments in rhesus macaque, using adenovirus as the vector, resulted in its immunization with antibody responses against S1 and T-cell responses against the N protein [68]. A recombinant vaccine constructed using a chimeric virus based on the vesicular stomatitis virus (VSV) with the G gene replaced by MERS-CoV S induced neutralizing antibodies and T-cell responses against MERS-CoV in rhesus monkeys after a single intramuscular or intranasal immunization dose [69]. Vaccination of rabbits with a recombinant fusion protein (RBD-Fc) containing 193 amino acid SARS-CoV RBD and human IgG1 Fc fragment led to induction of potent antibody response with complete inhibition of SARS-CoV infection [70]. Similarly, SARS-CoV-2-neutralizing antibodies were effectively induced in mice vaccinated with RBD-Fc developed using SARS-CoV-2 RBD [71]. Induction of humoral immune response and T- cell immunity was observed in albino rats vaccinated with recombinant NTD of the MERS-CoV S protein [72]. While T- cell responses are observed for both S and N proteins, it has been widely observed that neutralizing antibodies are directed only against the S protein, specifically, the RBD as the major immunodominant region [73,74]. Several subunit vaccines developed using peptide fragments of MERS-CoV RBD have induced robust immune responses in mice, specifically when administered by the intranasal route [75–78].

The economic viability, safety, effectiveness, and ease of rapid modification and production make synthetic peptides among the best antigenic determinants for the design and development of vaccines against viral pathogens [24]. However, the need for the viral peptide to be effectively presented by the MHC proteins and to invoke a subsequent B- and T-cell response makes selecting the candidate peptide most arduous. Specifically, the labor-intensive and expensive method of searching immunodominant epitopes by experimental evaluation of peptides from vast libraries fails the quick development of antiviral therapeutics during the ongoing pandemic [79]. A previous study on virus-specific cytotoxic T lymphocyte (CTL) immunity to HIV infection reported that individuals infected with the Human immunodeficiency virus (HIV) that do not progress to acquired immune deficiency syndrome (AIDS), have CTLs that target different MHC class I epitopes on HIV [80]. This observation suggests the advantages with *in silico* development of CTL vaccine for HIV and related viral diseases. Identification of several antigenic determinants has been achieved by prior predictions of B- and T-cell epitopes by bioinformatic analysis [79].

Interestingly, it has been reported that SARS-CoV-2 N protein contains multiple class I epitopes with predicted MHC restrictions that are consistent with broad population coverage [81]. A robust S protein-specific CD4^+^ T-cell reactivity in the majority of convalescing COVID-19 cases is congruous with the role of SARS-CoV-2 structural proteins in eliciting an adaptive immune response in the host [82]. Careful selection of specific immunogenic epitopes could help in the rational development of a multi-epitope peptide vaccine against any future β-CoVs. Several immune simulation studies involving integrated immunoinformatic approaches have designed such multi-epitope vaccines for inducing high levels of B- and T-cell mediated immunity (Figure 2) [83,84]. A peptide vaccine, EpiVacCorona, developed for protective immunity against SARS-CoV-2 has been approved after clinical trials [85]. This multi-epitope vaccine consists of synthetic fragments of SARS-CoV-2 S and N proteins that, upon administration, have been claimed to elicit an antibody response against the attachment and proliferation of the virus [86]. However, further studies and experimental validation of the designed multi-epitope peptide-based vaccines in inducing immune response and protection against β-CoV infections are necessary.

Vaccines have shown strong potency against SARS-CoV-2, with the major world population vaccinated with the BNT162b2, mRNA-1273, ChAdOx1, and BBV152 vaccines [87–90]. However, the emergence of SARS-CoV-2 variants with mutations in the spike protein has compelled the search for other immunotherapeutic candidates [91]. An *in silico* study examining the binding affinity of eight monoclonal antibodies (mAbs) against SARS-CoV-2 variants of Alpha and Delta lineages reported that regdanvimab, cilgavimab, and tixagevimab make stable complex formation with most Alpha strains; while sotrovimab, bamlanivimab, and tixagevimab showed neutralization of most Delta SARS-CoV-2 variants [91]. A chimeric antibody designed upon conjugation of CDRH3 regdanivimab with sotrovimab framework showed potential in preventing SARS-CoV-2 variants from escaping mAb-mediated neutralization [91]. Another study demonstrated the potential of using the antiparasitic drug Ivermectin for inhibition of SARS-CoV-2 protease, replicase, and human TMPRSS2 [92].

The candidate peptides that target the host cell<apos;>s translational machinery have demonstrated potent antiviral activity against β-CoV infections [93,94]. Ternatin-4, a fungal cyclic heptapeptide, is an inhibitor of eukaryotic translation elongation factor 1 A (eEF1A) that has demonstrated potential interactions with several β-CoV proteins and was reported to exert inhibition of SARS-CoV-2 (IC90 of 15 nM) in Vero E6 cells [95]. Another peptide candidate, Plitidepsin (cyclic depsipeptide isolated from *Aplidium albicans*), has been reported to directly interact and inhibit eEF1A [93]. The *in vivo* activity of Plitidepsin, used as a prophylactic treatment, has been associated with a two-fold reduction of SARS-CoV-2 replication in the lungs of mice [94]. Preclinical trials and randomized phase I studies of Plitidepsin against SARS-CoV-2 infected adults have reported a potent inhibition of Alpha, Beta, Delta, Mu, and Omicron variants, with a favourable safety profile in COVID-19 patients [93]. Furthermore, Plitidepsin was found to be more effective against both early and Alpha SARS-CoV-2 variants in human gastrointestinal and lung cell lines as compared to Remdesivir [93]. These immunotherapeutic peptides are potent candidates for β-CoV infections besides vaccines.

## 4. Peptide-based chemotherapeutics against β-CoVs

**Figure 3.**
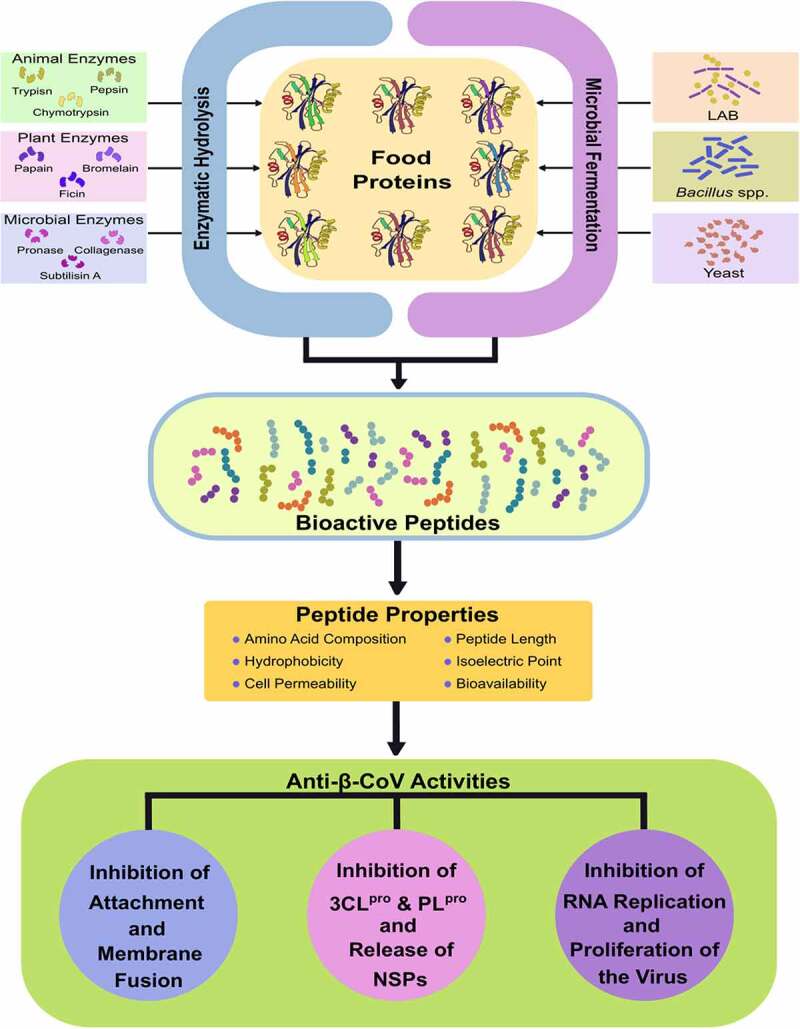
Potential anti-β-CoV activities of bioactive peptides released from food proteins by enzymatic hydrolysis and microbial fermentation. *In silico* analyses have demonstrated that food-derived peptides show potential inhibition of β-CoV attachment, entry, replication, and proliferation that is dependent on the amino acid composition, peptide length, bioavailability, and physicochemical properties of the peptides.

In addition to the extensive research on vaccines and immunotherapeutics against β-CoV, researchers around the globe are scouting for chemotherapeutics to combat the current pandemic and future β-CoV outbreaks [85,96]. These potential therapeutic solutions aim to target β-CoV infection, replication, and proliferation in addition to restoring the host<apos;>s immune response against the virus [97–99]. Rapid analysis of therapeutic targets against β-CoV and the design of potential drugs have been greatly achieved with the help of computational and bioinformatic methods [97,100]. Peptides are among the most explored candidates for anti-β-CoV therapeutic development due to higher levels of safety and effectiveness compared to small molecules [101–103]. Several studies have reported the β-CoV inhibitory potential of various natural, recombinant, and synthetic peptides (Figure 1 and Table 1) [22,98,104–108]. Antiviral peptides released upon microbial fermentation and enzymatic hydrolysis of food proteins have demonstrated potent inhibition of attachment and replication of β-CoVs during *in silico* studies (Figure 3). Despite these findings, there is a need for further in-depth study and extensive work on peptide-based candidates to develop effective therapeutics, specifically available against β-CoV infections.

**Table 1.** Inhibitory potential of natural and synthetic peptides against β-CoVs

Peptide sequence	Nature	Source organism/ product	Source Protein	Target β-CoV	Target protein/interaction	Inhibition stage	Reference
KFVPKQPNMIL	Natural	Soy cheese	Lectin	SARS-CoV-2, SARS-CoV, MERS-CoV, HCoV-HKU1	S1 RBD, 3CL^pro^	Attachment, Replication	[104]
PQQQF	Natural	*Hordeum vulgare*	D hordein	SARS-CoV-2	S1 RBD	Attachment	[115]
PISCR	Natural	*Triticum* sp.	Ribulose bisphosphate carboxylase	SARS-CoV-2	S1 RBD	Attachment	[115]
VQVVN	Natural	*Avena sativa*	11S globulin	SARS-CoV-2	S1 RBD	Attachment	[115]
VPW	Natural	*Tenebrio molitor*	Alpha-actinin-4	SARS-CoV-2	S1 RBD	Attachment	[105]
PW	Natural	*Cucurbita maxima*	Seed protein	SARS-CoV-2	S1 RBD, 3CL^pro^, PL^pro^	Attachment, Replication	[116]
ALNCYWPLNDYGFYTTTGIGYQPYRVVVLSFEL	Synthetic	SARS-CoV	S1 RBD	SARS-CoV	RBD-ACE2	Attachment	[120]
EEQAKTFLDKFNHEAEDLFYQSS-G-LGKGDFR	Synthetic	*Homo Sapiens*	ACE2	SARS-CoV	S RBD	Attachment	[121]
LGKGDFR	Synthetic	*Homo Sapiens*	ACE2	SARS-CoV-2	S1RBD	Attachment	[124]
NIQPPCRCC	Synthetic	*Moringa oleifera*	*Mo*-CBP3^a^	SARS-CoV-2	S1RBD	Attachment	[28]
GINASVVNIQKEIDRLNEVAKNLNESLIDLQELGKYE	Synthetic	SARS-CoV	S2 HR2	SARS-CoV	S2 HR1	Membrane fusion	[158]
SLTQINTTLLDLTYEMLSLQQVVKALNESYIDLKEL	Synthetic	MERS-CoV	S2 HR2	MERS-CoV	S2 HR1	Membrane fusion	[159]
LDLSDEMAMLQEVVKQLNDSYIDLKELGNYTYYNKW	Synthetic	Ty-BatCoV HKU4^b^	S2 HR2	MERS-CoV	S2 HR1	Membrane fusion	[127]
KAANRIKYFQ	Synthetic	*Arabidopsis thaliana*	Chitinase	SARS-CoV-2	S2 HR1	Membrane fusion	[28]
DISGINASVVNIQKEIDRLNEVAKNLNESLIDLQEL	Synthetic	SARS-CoV-2	S2 HR2	SARS-CoV-2	S2 HR1	Membrane fusion	[106]
SLDQINVTFLDLEYEMKKLEEAIKKLEESYIDLKEL-GSGSG-PEG4-Chol	Synthetic	HCoV-OC43	S2 HR2	SARS-CoV-2, MERS-CoV, HCoV-OC43	S2 HR1	Membrane fusion	[107]
AVLQSGFR	Synthetic	SARS-CoV	3CL^pro^	SARS-CoV	3CL^pro^	Replication	[133]
EEAGGATAAQIEM	Natural	*Thunnus thynnus*	Skeletal myosin	SARS-CoV-2	3CL^pro^	Replication	[22]
RVCGVSAARLTPCGTG	Synthetic	SARS-CoV-2	3CL^pro^	SARS-CoV-2	3CL^pro^	Replication	[108]
Ac-hT-Dap-G-G-VME	Synthetic	SARS-CoV	PL^pro^	SARS-CoV, SARS-CoV-2	PL^pro^	Replication	[140]
HXAWFK	Synthetic	*Homo Sapiens*	Ghrelin	SARS-CoV-2	RdRp	Replication	[147]
GGASCCLYCRCH	Synthetic	SARS-CoV	NSP10	SARS-CoV	2’-O-MTase^c^	Replication	[144]
YGGASVCIYCRSRVEHPDVDGLCKLRGKF	Synthetic	MHV	NSP10	SARS-CoV, MERS-CoV	2’-O-MTase	Replication	[145]

*Mo*-CBP3 – *Moringa oleifera-* chitin binding protein 3; Ty-BatCoV HKU4 – Tylonycteris bat Coronavirus HKU4; 2’-O-MTase – 2’-O-methyltransferase.

### 4.1 Food derived peptides as potential therapeutics against β-CoVs

Food-derived peptides have demonstrated interaction with β-CoV structural proteins and NSPs that may prevent viral infection and proliferation [28,104]. Bioactive peptides released upon fermentation of foods exert several functionalities that include antimicrobial, antioxidant, antihypertensive, and anticancer properties and can be explored to develop nutraceuticals and therapeutics (Figure 3) [109–112]. Fermented food-derived peptides have previously demonstrated high antiviral activity against viral pathogens [113]. The peptide, KFVPKQPNMIL, derived from soy cheese produced using *Lactobacillus delbrueckii* WS4, demonstrated high binding affinity towards key residues of both S1 RBD and 3CL^pro^ of SARS-CoV-2, SARS-CoV, MERS-CoV, and HCoV-HKU1, thereby indicating a potential for inhibition of both attachment and replication of β-CoVs [104]. Such food-derived peptides could potentially bind with multiple viral proteins could be used as lead compounds to develop potent therapeutics against β-CoVs. Molecular docking studies of peptides derived from fermented soybeans against SARS-CoV-2 RBD and human TLR4/Myeloid Differentiation factor 2 (MD2) complex revealed that the peptide ALPEEVIQHTFNLKSQ, generated during soybean fermentation with *Bacillus licheniformis* KN1G showed a high binding affinity with both S1 RBD and TLR4/MD2 complex [114]. This study indicated the peptide<apos;>s potential in inhibiting viral attachment and regulation of cytokine storm induced by SARS-CoV-2 [114].

High-affinity binding with SARS-CoV-2 S1 RBD was observed during molecular docking studies using peptides obtained from *in silico* gastrointestinal (GI) digestion of wheat, barley, and oat proteins [115]. In another *in silico* study, the peptide VPW, derived from edible mealworms showed a superior binding affinity with SARS-CoV-2 RBD as compared to some natural products [105]. *In silico* GI digestion of storage proteins from quinoa, sesame, rape, sunflower, and pumpkin seeds resulted in the release of several peptides with high GI absorption that demonstrated binding affinities towards multiple structural proteins and NSPs of SARS-CoV-2 during molecular docking studies [116]. Peptides generated upon i*n silico* GI digestion of marine fish proteins have demonstrated a high affinity for key catalytic residues of SARS-CoV-2 3CL^pro^ [22,117]. The tuna skeletal myosin-derived peptide EEAGGATAAQIEM demonstrated good water solubility, no toxicity, and high binding affinity for critical residues of 3CL^pro^, including the HIS41-CYS145 catalytic dyad [22]. Such food-derived peptides, capable of inhibiting viral entry and replication, can be used to develop therapeutics and prophylactics against current and future β-CoV diseases [118].

### 4.2 Synthetic peptide and peptide-based therapeutics against β-CoVs

The emergence of several novel synthetic strategies has empowered the design and modification of peptides that offer desired therapeutic functionality against a broad spectrum of viral pathogens [85]. Synthetic peptides are cheap, easy to mass-produce, and highly pure as compared to natural or recombinant peptides [119]. Several studies have reported the antiviral activity of peptides derived from structural proteins and NSPs of β-CoVs, and host cell receptor proteins making them favorable candidates for the development of antiviral therapeutics [85].

#### 4.2.1 Inhibitors of RBD-receptor interaction and membrane fusion

Impeding the interaction between S1 RBD and the receptor protein can inhibit the attachment of the virus to its host cell. Peptides derived from the RBD of β-CoVs can competitively bind to the receptor protein and exert antiviral activity. A SARS-CoV RBD-derived peptide (amino acids 471–503) specifically blocked RBD-ACE2 interaction, resulting in the inhibition of entry of SARS-CoV into Vero cells with an IC_50_ of approximately 40 μM [120]. In another study, a chemically synthesized polypeptide, containing two RBD binding motifs of ACE2, was artificially linked together by glycine leading to potent inhibition of SARS pseudovirus infection in HeLa cells with an IC50 of 0.1 μM [121]. Studies have reported the inhibition of MERS-CoV entry into host cells using neutralizing mouse mAbs [122]. These mAbs were generated by immunizing mice with synthetic peptide complexes derived from MERS-CoV spike protein [123]. Molecular docking and dynamic simulation studies of numerous synthetic peptides, based on sequences derived from ACE2 protease domain and human antimicrobial peptides, have revealed specific and stable binding with SARS-CoV-2 S1 RBD [28,96,124]. The absence of side effects, such as hemolytic activity, toxicity, and the superior binding affinity for spike protein over ACE2, increase the favourability of using peptides to develop therapeutics against SARS-CoV-2 attachment without interfering with ACE2 activity [28].

Inhibition of the fusion of viral spike protein with the host cell membrane has been achieved by synthetic peptides derived from the HR2 region in the S2 domain of the spike protein, which competitively binds with the HR1 domain and blocks the formation of the fusion core [85]. The fusion peptide inhibitors derived from regions of S2 protein outside the fusion protein heptad repeats, such as the N-terminal or the pre-transmembrane domain of the SARS-CoV S2 protein, have shown potential as antiviral agents [125]. SARS-CoV-2 demonstrates a significantly higher membrane fusion capacity than SARS-CoV; therefore, the development of SARS-CoV-2 fusion inhibitors is of significant value [107]. Several fusion inhibitory peptides derived from the HR2 domain, such as MERS-HR2P from MERS-CoV HR2 and CP1 from SARS-CoV HR2, have been previously reported [126–129]. Synthetic HR2-based peptides designed by molecular dynamics simulation of the SARS-CoV-2 fusion core have demonstrated a stronger binding with HR1 as compared to the natural stage of the fusion core [106]. Researchers have recently designed HR2-based lipopeptides with the ability to inhibit the fusion of SARS-CoV-2 to the target cell [107,130]. EK1C4, a lipopeptide developed by conjugating a cholesterol molecule to the pan-coronavirus fusion inhibitor peptide EK1, exhibited a 240- and 150-fold higher inhibitory activity against SARS-CoV-2 S2-mediated membrane fusion and pseudovirus infection, respectively [107]. EK1C4 demonstrated a high fusion inhibitory activity against *in vitro* and *in vivo* infection of live SARS-CoV-2, HCoV-OC43, and MERS-CoV, suggesting the potential of using peptide-based fusion inhibitors for the development of therapeutics against pan-β-CoV infections [107].

#### 4.2.2 Peptides targeting β-CoV NSPs

The two cysteine proteases, 3CL^pro^ and PL^pro,^ are conserved among major β-CoV human pathogens, SARS-CoV, MERS-CoV, and SARS-CoV-2 among the most critical drug targets for developing therapeutics [17,131,132]. The synthetic octapeptide, AVLQSGFR, forms strong hydrogen bonds with catalytic residues of SARS-CoV 3CL^pro^, actively inhibiting the replication of SARS-CoV in Vero cells [133]. Similarly, several synthetic peptides have been proposed to inhibit replication of several β-CoV strains by blocking the activity of the 3CL^pro^ protein [134–138]. Synthetic peptides designed using computational models have shown strong binding affinity against SARS-CoV-2 3CL^pro^ [108,139]. Synthetic evolutionary peptides were designed using machine learning algorithms based on conserved 3CL^pro^ motifs from diverse viral sequences of COVID-19 cases reported from Italy, the USA, India, and China [108]. Four peptides from the designed library showed strong and stable binding affinities against SARS-CoV-2 3CL^pro^ [108]. Likewise, inhibitory peptides, designed with a high degree of selectivity for SARS PL^pro^ have demonstrated a high binding affinity for critical residues of SARS-CoV-2 PL^pro^ [140].

The 2’-O-methylation of the viral mRNA cap, catalyzed by the 2’-O-methyltransferase (2’-O-MTase) enzyme, NSP16 of β-CoVs, serves as a molecular signature for the differentiation of self mRNA from host mRNA, which helps the virus to evade host immune systems [141,142]. A class of zinc finger protein, NSP10, interacts with NSP16 and this interaction is crucial for 2’-O-MTase activity of NSP16 [143]. The inhibition of NSP16 can lead to the suppression of viral replication and the prevention of viral infection. Short synthetic peptides derived from the interaction domain of NSP10 demonstrated (*in vitro*) inhibition of SARS-CoV NSP10/NSP16 complex activity [144]. Similarly, the peptide P29, YGGASVCIYCRSRVEHPDVDGLCKLRGKF, derived from NSP10 of mouse hepatitis virus (MHV), demonstrated 2’-O-MTase inhibitory activity against SARS-CoV and MERS-CoV, with an inhibitory efficiency of >50% [145].

Researchers studying inhibition of SARS-CoV-2 RdRp have mainly focussed on existing antiviral drugs owing to the advantages of repurposing strategies that build on previous research, the candidate drug is ready for clinical trials. It can be quickly approved by the food and drug administration (FDA) [146]. Nucleoside analogues, including Remdesivir, Favipiravir, and Ribavirin, have shown potent *in vitro* RdRp inhibitory activity and have entered clinical trials [147]. However, some participants’ decrease in the inhibitory activity and the emergence of adverse effects, including hepatotoxicity, respiratory toxicity, cardiovascular toxicity, nephrotoxicity, reproductive toxicity, and gastrointestinal symptoms, have prevented the approval of nucleoside analogues for use in COVID-19 patients [148]. Peptide-based inhibitors against RdRp can overcome such adverse effects due to the safety profile of peptide therapeutics. Interestingly, in molecular docking studies, the FDA-approved synthetic peptide drug Examorelin showed strong binding efficacy with both core and holoenzyme of SARS-CoV-2 RdRp [147]. Clinical trials of such peptide-based candidates can lead to the development of anti-β-CoV therapeutics with minimum or nil risk of adverse effects [149]. However, the use of advanced biotechnological tools for increasing peptide bioavailability, corroborated by *in vivo* studies, is necessary for developing peptide therapeutics against β-CoV [101,150]. A recent study has reported that ACE2, TMPRSS2, and TMPRSS4 of tree shrew are more similar to humans (85.47%) as compared to rats (82.58%) and mice (82.81%), suggesting the potential use of tree shrew models for *in vivo* investigations of peptide therapeutics against β-CoV infections [40].

## 5. Using CPPs as intracellular shuttling vectors of anti-β-CoV therapeutics

The hydrophobic nature of the cell membrane acts as a major obstacle for drug delivery, resulting in a reduced potency of therapeutics. Both naturally derived and synthetic CPPs have been extensively investigated as carriers of membrane-impermeable molecules for intracellular drug delivery [151]. CPPs deliver the cargo therapeutic through caveolae-mediated endocytosis, micropinocytosis, or the clathrin-independent endocytosis mechanism [152]. The well-known CPP, HIV-1 Tat (RRRQRRKKR), was used for intracellular transportation of antisense peptide nucleic acids that inhibit ribosomal frameshifting resulting in the suppression of SARS-CoV replication [151]. Similarly, CPPs can be used for the intracellular transportation of therapeutic drugs targeted to suppress SARS-CoV-2 replication while maintaining the potency of the drug<apos;>s inhibitory activity.

Since viruses are intracellular obligate parasites, a large number of CPPs originating from viruses have been used as intracellular shuttling vectors to facilitate the transportation of cargos through the host cell membrane [30]. CPPs have several advantages over other drug delivery methods, such as a high rate of cellular permeability, higher uptake capacity, reduced cell toxicity, the capability to translocate into a diverse range of cell types, and an easy and inexpensive production process [152]. Interestingly, four novel CPPs, SCV2-CPP118, SCV2-CPP119, SCV2-CPP122, and SCV2-CPP129, have recently been identified from SARS-CoV-2 RdRp, based on *in silico* evaluation of physiochemical properties, protease susceptibility, uptake efficiency, membrane interaction, higher helical or sheet secondary structures, and toxicity [30]. These peptides can be used as drug delivery vectors for therapeutics against replication of SARS-CoV-2 and other β-CoV pathogens. However, *in vivo* analysis of the drug-carrying capacity of these CPPs is necessary, including biotechnological modification of the peptides to overcome potential CPP drawbacks such as metabolic instability, probable allergenicity, proteolytic cleavage, and endosomal entrapment and degradation [30].

## 6. Challenges associated with the development of peptide-based therapeutics

High selectivity, efficiency, safety, and tolerability of peptides have piqued the researchers for the development of prudent and potent therapeutics [19]. The discovery of anti-β-CoV activities of several natural and synthetically designed peptides, targeting attachment and replication of the virus, cements the requirement of peptide-based prophylactics and therapeutics against COVID-19 future pandemics. However, the development of peptide-based therapeutics suffers certain potential drawbacks, including chemical and physical instability, susceptibility to proteolytic hydrolysis, a tendency for aggregation, and low bioavailability and membrane permeability of peptides [19,101,153].

Several strategies have been proposed over the years to overcome the barriers of peptide therapeutic developmental efforts. Alteration of both the amide bond and the side-chains can result in peptidomimetics that are resistant to proteolytic degradation [154]. The introduction of D-amino acids in the peptide leads to cyclization that confers the peptide resistance against proteolytic degradation and increases absorption after oral administration [101,154]. For peptides not amenable to cyclization, attachment of polyethylene glycol (PEG) chains increases absorption and systemic stability of the peptide therapeutic [155]. Cell penetration of peptide therapeutics can be improved by adding positively charged amino acids at terminal positions to facilitate passive or active transport of the peptides through membranes [156]. CPPs contain several positively charged amino acids and are widely used for the delivery of various therapeutics [157]. CPPs derived from the SARS-CoV-2 proteome can be used to efficiently deliver peptide therapeutics against COVID-19 and related diseases [30]. Alternatively, conjugation of therapeutic peptides to ligands of cell surface receptors, including cell adhesion receptors, carbohydrate receptors, lipoprotein receptors, and transferring receptors, can facilitate better internalization of peptide therapeutics [153]. Administration by alternate delivery routes, such as intranasal delivery of pan-β-CoV fusion inhibitory peptide EK1C4, increases the stability and bioavailability of peptide therapeutics [107]. The inclusion of such strategies can help to develop safe, efficient, and effective peptide-based prophylactics and therapeutics against present and future β-CoV associated diseases.

## 7. Concluding remarks and future perspectives

Studies have reported a wide variety of anti-β-CoV activities of peptides over the past two decades. Specific peptides could be synthesized to develop vaccines and therapeutics that are effective against mutating viral pathogens. However, large-scale peptide synthesis is expensive. Specific challenges need to be addressed before achieving peptide-based therapeutics. The β-CoV inhibitory activities of many peptides have been reported by *conducting in silico* simulation studies. It is vital to validate the therapeutic activities of these peptides by *in vitro* and *in vivo* studies. The relatively large size of peptides makes them susceptible to proteolytic degradation, resulting in low bioavailability and short half-lives of peptide-based drugs. However, several modification strategies can improve the stability and activity of therapeutic peptides. In-depth research is required to design potent peptides with superior efficacy and bioavailability. Peptide therapeutics are promising to combat β-CoV pathogens and related viral diseases.
